# Biophysics of lumen morphogenesis

**DOI:** 10.1242/dev.205628

**Published:** 2026-08-24

**Authors:** Byung Ho Lee, Markus Mukenhirn, Tristan Guyomar, Yann Maggipinto, Sakurako Tanida, Kana Fuji, Linjie Lu, Felix Romer, Makiko Nonomura, Tetsuya Hiraiwa, Masaki Sano, Alf Honigmann, Daniel Riveline, Anne Grapin-Botton

**Affiliations:** ^1^ Max Planck Institute of Molecular Cell Biology and Genetics, Dresden 01307, Germany; ^2^ Technische Universität Dresden, Biotechnologisches Zentrum, Center for Molecular and Cellular Bioengineering (CMCB), Dresden 01307, Germany; ^3^ Institut de Génétique et de Biologie Moléculaire et Cellulaire, Illkirch 67404, France; ^4^ Université de Strasbourg, Illkirch 67404, France; ^5^ Centre National de la Recherche Scientifique, UMR7104, Illkirch 67404, France; ^6^ Institut National de la Santé et de la Recherche Médicale, U964, Illkirch 67404, France; ^7^ Universal Biology Institute, Graduate School of Science, The University of Tokyo, Tokyo 113-0033, Japan; ^8^ Department of Aeronautics and Astronautics, Graduate School of Engineering, The University of Tokyo, Tokyo 113-8656, Japan; ^9^ Department of Mathematical Information Engineering, College of Industrial Technology, Nihon University, Chiba 275-8576, Japan; ^10^ Institute of Physics, Academia Sinica, Taipei 11529, Taiwan; ^11^ Mechanobiology Institute, National University of Singapore, Singapore 117411; ^12^ Technische Universität Dresden, Biotechnologisches Zentrum, Center for Molecular and Cellular Bioengineering (CMCB), Dresden 01307, Germany; ^13^ Cluster of Excellence Physics of Life, TU Dresden, 01062 Dresden, Germany

**Keywords:** Lumen, Epithelia, Extracellular matrix, Organogenesis, Biological physics

## Abstract

Although lumen formation is a key feature of organogenesis, luminal compartments – formed through the secretion of fluid, ions and macromolecules by the cells, usually on the apical side – are often viewed as passive by-products of epithelial polarity. Here, we discuss how luminal fluids actively contribute to morphogenesis in metazoans through mechanical, hydraulic and electrochemical interactions with the surrounding epithelia and the extracellular matrix (ECM). We first outline the diversity of lumen architectures and summarize conserved mechanisms of lumen nucleation, fusion, folding and growth across metazoans. We then present the physical principles governing lumen morphogenesis, highlighting how hydrostatic and osmotic pressures, cortical tension and matrix mechanics interact to shape tissues. Furthermore, theoretical modeling and numerical simulations provide a quantitative framework for predicting phenotypes related to lumen shape, exploring the parameter space of hydraulic and active tissue mechanics variables, and linking molecular perturbations to emergent tissue geometries. Finally, we examine feedback loops whereby lumen-associated forces regulate junctional integrity, proliferation, differentiation and tissue patterning. Together, these concepts position luminal fluids as active regulators of lumen morphogenesis.

## Teaser

**Summary:** This Review explains the interplay between lumina, the surrounding tissue and the ECM from the perspective of both developmental biology and physics.

## INTRODUCTION

Developmental biology has traditionally emphasized gene regulation, signaling pathways and cell-intrinsic programs that drive morphogenesis; however, tissues do not develop in isolation. Mechanobiology has established that reciprocal interactions between cells and their physical environment are essential for the emergence of tissue architecture and fate decisions (Alasaadi and Mayor, 2024; De Belly et al., 2022).

In metazoan epithelial tissues, cells exhibit apical–basal polarity, with their apical surface of cells typically facing fluid environments and their basal surface anchored to the ECM. Together, these apical and basal compartments provide biochemical, mechanical and physical cues that enable asymmetries in cell behavior. One key consequence of epithelial polarity is the directed secretion of fluid into the intercellular space, typically from the apical domain, that results in lumen formation (Chan and Hiiragi, 2020).

These fluid-filled lumina, which usually form at the apical domain of epithelia and are sealed by tight junctions between neighboring cells, constitute a central component of the physical environment that is essential for cell fate decisions and organogenesis (Sigurbjornsdottir et al., 2014). Across metazoan embryos and organ systems, luminal cavities harbor osmotic gradients, hydrostatic pressure and bioelectrical fields that feedback on epithelial mechanics, growth and patterning. Despite the abundance of fluid-filled lumina in many tissues, their functions are only beginning to be fully appreciated. However, emerging studies have expanded our understanding of their functions and have begun to shed light on the mechanisms that control the diversity of lumen shapes during normal and pathological development.

In this Review, we synthesize the current understanding of how cavity-enclosed fluids contribute to morphogenesis. Although lumina are also found in plants and can form within single cells, we focus on metazoan-specific extracellular lumen morphogenesis and associated tissue mechanics. Lumen formation is an evolutionarily ancient process that has even been observed transiently in multicellular colonies of ichthyosporeans (Araújo et al., 2026 preprint). Here, we first describe the diversity of lumen architectures and functions, as well as the biogenetic routes through which they arise, including coordinated cell polarization, the deposition of cell-derived material within a shared expanding cavity and subsequent shape remodeling. We then discuss the physical principles governing lumen–epithelium–ECM interactions, alongside computational frameworks used to predict and quantitatively study phenotypes related to the disruption or failure of normal lumen formation. As this Review aims to combine molecular and physical insights within a single framework, we provide focused, rather than exhaustive, coverage of each field and direct the reader to complementary reviews, where relevant (Bryant and Mostov, 2008; Torres-Sanchez et al., 2021). Finally, we examine feedback mechanisms through which luminal forces regulate proliferation, differentiation and tissue organization across metazoan species, highlighting unresolved questions and future directions.

## Biogenesis and functions of luminal cavities

### Diverse morphologies and functions of lumina

**Fig. 1.**
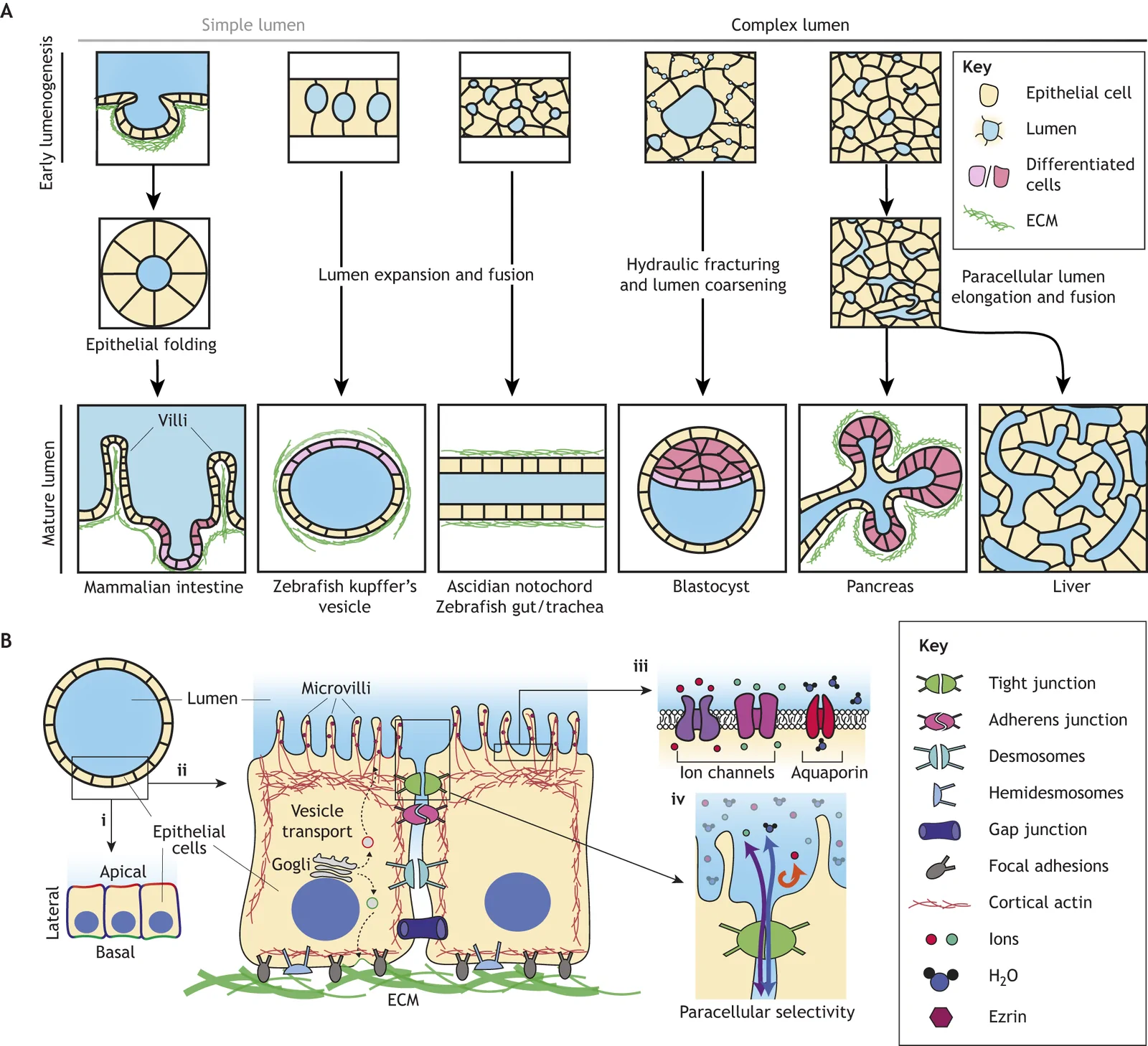
**Lumen architecture, formation, and cellular and molecular components.** (A) Schematic overview of examples of lumen organization across epithelial tissues across different models of vertebrate embryonic development, as well as in the ascidian notochord. The top panel illustrates lumen initiation in monolayered and pseudostratified epithelia. Distinct morphogenetic routes are depicted, including folding, lumen expansion and fusion, hydraulic fracturing and lumen coarsening, and paracellular lumen elongation and fusion. Representative tissue and lumen examples are shown below: mammalian intestine, zebrafish Kupffer's vesicle, ascidian notochord and zebrafish gut/trachea, mouse blastocyst, the pancreas, and the liver, highlighting spherical, tubular, branched and networked lumen geometries. (B) Subcellular organization of a polarized epithelial cell lining a lumen. (i) Cells display apical–basal polarity, with an apical surface facing the lumen and a basal surface contacting the extracellular matrix (ECM). (ii) The actin cortex spans the plasma membrane and connects laterally to neighboring cells via tight junctions, adherens junctions, desmosomes and gap junctions. Integrin-mediated adhesions anchor cells basally to the ECM. Apical microvilli project into the lumen and are coupled to the cytoskeleton via ezrin, radixin (not shown) and moesin (not shown). These anchors enable the application of cytoskeleton-generated forces on the neighboring cells, the ECM and the lumen. They also enable the sensing of forces from the environment. (iii) Ion channels and aquaporins regulate transcellular ion and water transport, whereas (iv) paracellular permeability between cells modulates fluid flux. They play an important role in lumen dynamics by retaining or leaking water and ions, which interact with lumen matrix proteins. Together, these components contribute to force balance at each point and lumen shape.

The formation and organization of luminal spaces within epithelial tissues is a key process in the development of organs, known as organogenesis. Lumina are fluid-filled cavities that vary widely in shape and function across species and organ systems (Fig. 1A). For example, the thyroid gland contains spherical sacs, the gastrointestinal tract forms a tube with surface-enlarging villi, and the kidney, the vasculature and the pancreas develop complex networks of interconnected tubes (Camelo and Luschnig, 2021; Flasse et al., 2021; Goodwin and Nelson, 2020; Pierreux, 2021). Lumina can also acquire more-complex shapes, such as those found in the mammalian inner ear (Mosaliganti et al., 2019; Munjal et al., 2021) and heart (Gunawan et al., 2021).

Beyond their diverse morphologies, lumina serve diverse physiological functions: they can act as storage compartments (thyroid and bladder), as conduits for fluid exchange (lungs, vasculature, kidney and Malpighian tubes) and nutrient distribution (gut), as sensory compartments (Kupffer vesicle, lateral line canal and otic vesicle), and as hydraulic skeletons (polyp coelenteron and penis cavernous corpse), cushions (coelom and placenta) or locomotory systems (echinoderm tube feet) (Kier, 2012; Stokkermans et al., 2022).

### Overview of lumenogenesis steps

At the cellular level, lumenogenesis depends on epithelial polarization, which establishes distinct apical (usually lumen-facing) and basal (matrix-facing) domains (Fig. 1Bi). In rare cases, this polarity can be inverted, such as in the mammalian blastocyst (Maitre, 2017). This apical–basal polarity is coordinated and maintained by vesicle transport, and directs spatial asymmetries in ion pumping, cortical actin stress generation and cell division (Camelo and Luschnig, 2021). Tight and adherens junction complexes play crucial roles in maintaining the structural integrity of the lumen by regulating epithelial permeability and barrier function through tight junctions, and by generating supracellular actin-based tension through both adherens and tight junctions. While current studies have provided foundational insights into cell polarization, lumen initiation and growth, we are far from understanding the variability and complexity of lumen formation across different tissues (Camelo and Luschnig, 2021).

Multiple mechanisms contribute to luminal shape (Fig. 1A), including the location of multiple nucleation sites arising either at the midbody after cell division, between initially unpolarized cells or even within single cells (Sigurbjornsdottir et al., 2014). Lumina can also form through diverse biogenetic mechanisms, including: cavitation, where programmed cell death contributes to lumen formation (Datta et al., 2011); cord hollowing, whereby multiple cells establish and coordinate their apicobasal polarity (Strilic et al., 2009); epithelial folding, as observed in the *Drosophila* trachea or salivary gland (Abrams et al., 2003; Hayashi and Dong, 2017); and single cell wrapping or hollowing mechanisms. These diverse modes of lumen formation underline the importance of studying how the general principles of lumen formation, including conserved physical and cellular mechanisms, are adapted to their tissue**-**specific context.

#### Lumen nucleation

Lumen nucleation refers to the initial emergence of a *de novo* apical space within a cell cluster or tissue, marking the transition from a previously unpolarized epithelium to one with an established apical domain and a nascent luminal cavity (Fig. 1A). The step of lumen nucleation is therefore relevant to lumen formation by cord hollowing and cavitation, but not to lumen formation by epithelial folding or single cell wrapping mechanisms. *In vivo*, lumen nucleation has been observed in diverse developmental systems, including the early mouse blastocyst (Dumortier et al., 2019) (Fig. 1A) and epiblast (Bedzhov and Zernicka-Goetz, 2014), the zebrafish gut tube (Horne-Badovinac et al., 2001) (Fig. 1A), the mouse salivary gland (Nedvetsky et al., 2014), the pancreas (Kesavan et al., 2009; Villasenor et al., 2010) (Fig. 1A), and the developing vasculature and heart (Medioni et al., 2008; Strilic et al., 2009). Lumina are also nucleated in invertebrates such as the *C. elegans* intestine (Leung et al., 1999) and the *Hydra* or the *Nematostella* gastrovascular cavity (Fütterer et al., 2003; Kucken et al., 2008; Stokkermans et al., 2022; Suzuki et al., 2023 preprint).

Madin-Darby canine kidney (MDCK) cell cysts remain one of the best-characterized *in vitro* mammalian models of lumen nucleation. When cultured as three-dimensional (3D) cysts within ECM scaffolds such as Matrigel, MDCK cells recapitulate polarity establishment and lumen initiation on the apical side (Bryant et al., 2010; Overeem et al., 2015). However, studies using the MDCK model have also shown that, in the absence of an ECM scaffold, cells cultured in suspension can form a lumen on the basal side (Wang et al., 1990). This inverted polarity has also been confirmed in intestinal organoids (Co et al., 2019) and in some tumor organoids (Zajac et al., 2018), and is relevant to the blastocyst basal lumen formation *in vivo* (Dumortier et al., 2019; Maitre, 2017) as well as in the blood vessels of some invertebrates (Kucera et al., 2009; Reiber and McGaw, 2009). In MDCK cells, a crucial step is the re-localization of apical proteins (Fig. 1B), such as podocalyxin, which are initially exposed to the ECM but are subsequently trafficked toward an apical membrane initiation site. This site is defined by polarity proteins that assemble around the midbody, where the first luminal surface forms by the fusion of apical vesicles (Bryant et al., 2010; Desclozeaux et al., 2008; Klinkert et al., 2016; Lu et al., 2025). As MDCK cysts grow, coordination of the cell division plane with the polarity machinery ensures that newly formed apical membranes at the midbody integrate with and expand the existing apical domain, thereby promoting the growth of a single lumen (Martin-Belmonte et al., 2008). The MDCK model has been crucial in advancing our knowledge of the molecular and mechanical aspects of lumen nucleation in mammalian systems by providing a simplified *in vitro* environment in which perturbations can be easily introduced, while *in vivo* models further reveal how these mechanisms contribute to the emergence of tissue-specific lumen shapes.

#### Lumen fusion

Although some organs can generate complex lumina through asymmetric growth of a lumen, lumen fusion is a frequent process wherein several separate luminal cavities, often formed asynchronously, subsequently merge to form one continuous lumen. This requires cells to remodel their epithelium, remove the junctions separating adjacent cavities, align their apical surfaces and enlarge the cavities through fluid-driven expansion bringing lumina into contact. This mechanism is broadly utilized by several organs: for instance, in the zebrafish intestine, brain, Kupffer vesicle (Fig. 1A) and inner ear, multiple microlumina first open independently and then resolve into a single lumen (Alvers et al., 2014; Hoijman et al., 2015; Lowery and Sive, 2005; Navis et al., 2013). Fusion-type mechanisms also contribute to posterior mammalian neural tube formation, where secondary neurulation proceeds by cavitation within a medullary cord to generate a single continuous lumen (Saade and Marti, 2025). A similar process occurs during notochord hollowing in ascidians, where the fusion of nascent luminal spaces contributes to the establishment of a continuous axial cavity (Fig. 1A) (Deng et al., 2013; Denker et al., 2015). In the mammalian pancreas, multiple initially small lumina coalesce into a unified luminal network (Fig. 1A) (Kesavan et al., 2009; Villasenor et al., 2010), a process that has been recapitulated in pancreatic organoids under conditions of low pressure and high proliferation (Lee et al., 2026). Finally, although not a fusion event in the strict sense, in the mammalian blastocyst, a field of microlumina generated by hydraulic fracturing undergoes active coarsening, with fluid progressively draining into one dominant cavity – the blastocoel (Fig. 1A) (Dumortier et al., 2019; Ryan et al., 2019; Schliffka et al., 2024). Finally, this growth and fusion mechanism between cysts was also recently reproduced with the MDCK model (André et al., 2026).

In addition to the above examples of fusion between pre-existing extracellular lumina, lumen formation can also involve the fusion of intracellular lumina that arise within individual cells before connecting with neighboring cells or tissues. For example, during *Drosophila* tracheal development, intracellular lumina form within individual epithelial cells and subsequently fuse to generate a continuous tubular network, contributing to the establishment of the respiratory system (Samakovlis et al., 1996). Similarly, in the zebrafish vasculature, endothelial cells can generate intracellular luminal compartments that later merge as cells interconnect during the formation and remodelling of blood vessels (Campinho et al., 2020).

#### Lumen growth and folding

Lumen growth begins after nucleation. While lumen fusion events contribute to promoting lumen growth, vesicular fusion provides apical membranes and luminal material, while ion secretion and fluid accumulation drive volume growth. In addition, cell division and changes in individual cell aspect ratios can expand their apical sides (Guha Ray et al., 2025; Lu et al., 2025; Mukenhirn et al., 2024; Tanida et al., 2025). Depending on geometry and tissue mechanics, this expansion can be isotropic, producing a spherical lumen, or anisotropic, generating elongated or branched structures (Fig. 1A). During growth, the ratio of apical membrane to volume growth can vary depending on the shape of the lumen; this ratio decreases by water influx, but increases through vesicle fusion. In addition, actin polymerization can contribute to driving lumen growth (Indana et al., 2024).

By contrast, anisotropic growth biases expansion along a preferred axis to form tubes and networks. This mode of expansion underlies the formation of the *Drosophila* tracheal system, where signaling and cell rearrangements orient axial elongation and size control (Forster and Luschnig, 2012; Hayashi and Dong, 2017). In vertebrate blood vessels, endothelial tubes extend and remodel along the vascular tree under flow-derived mechanical cues that polarize, migrate and reshape endothelial cells during network morphogenesis (Campinho et al., 2020; Strilic et al., 2009). Glandular organs such as the pancreas use repeated lumen initiation, growth and remodeling to generate branched anisotropic lumina, forming an interconnected network of multi-lumen tubes, which is then progressively refined into a single-lumen ductal tree (Jackson et al., 2026; Dahl-Jensen et al., 2018; Flasse et al., 2021; Villasenor et al., 2010). Furthermore, mouse lung and kidney epithelia exhibit biased (anisotropic) tube elongation in early development, where apical shear stresses from luminal flows can explain directional growth and cell-shape bias (Conrad et al., 2021).

Beyond expansion, lumen morphogenesis also involves folding, whereby an existing epithelial cavity can be initiated or is reshaped through buckling, bending, invagination or evagination (Tozluoǧlu and Mao, 2020). Folding reorganizes epithelial sheets via differential growth, apical constriction, basement-membrane tension or hydrostatic pressure. The lumen of the vertebrate otic vesicle (Mosaliganti et al., 2019; Munjal et al., 2021), gut tube (Gill et al., 2024) and spinal cord central canal (Saade and Marti, 2025), as well as the *Drosophila* trachea (Samakovlis et al., 1996) and salivary glands (Abrams et al., 2003; Gillard and Roper, 2024) are some examples where lumen formation is initiated by folding. In the intestine, patterned folding of the initial tube generates crypt–villus architecture (Shyer et al., 2013; Yavitt et al., 2023) and organoids recapitulate crypt budding through curvature-inducing mechanical asymmetries and mechanochemical feedback (Xue et al., 2025; Yang et al., 2021).

### Role of the ECM in lumenogenesis

In addition to the processes occurring within the epithelial lining that are crucial for lumen growth and shape morphogenesis, the ECM produced by the epithelial cells or their surrounding mesenchyme plays a crucial role in shaping luminal architecture. The ECM can be deposited in the lumen or on the basal side of epithelial cells (Loganathan et al., 2020) and has important functions in tissue mechanics (Saraswathibhatla et al., 2023).

The apical matrix often consists of intertwined porous elastic networks that are charged, generating repulsive electrostatic lumen-enlarging force. They also bind ions and organize water molecules, generating swelling forces that push outward against the epithelial boundary. The involved polymers include membrane-anchored or secreted glycoproteins such as mucins (Syed et al., 2012, 2022), which regulate lumen size in the gut tube, lungs, diverse glands, kidney and blood vessels (Strilic et al., 2009; Syed et al., 2022). Several proteoglycans control lumen size and shape in the *Drosophila* hindgut foregut, proventriculus, salivary gland ducts, tracheal dorsal trunks (Syed et al., 2012) and eye rhabdomere lumen (Husain et al., 2006), as well as in the *C. elegans* vulva (Cohen et al., 2020) and the hearts of multiple species (Jahnel et al., 2026 preprint; Medioni et al., 2008). These molecules usually expand but can also constrict lumen shape (Cohen et al., 2020). They can additionally promote folding, as seen in the sea urchin archenteron (Lane et al., 1993). Glycoproteins can be secreted into the side of the cells opposite to the lumen; e.g. in the blastocyst, apical ECM components are on the apical side of the trophectoderm, opposite to the basal blastocoele and thereby constrain lumen enlargement (Chan et al., 2019; Leonavicius et al., 2018).

In addition, rigid fibrous molecules such as the polysaccharide chitin (Devine et al., 2005; Tonning et al., 2005), together with associated proteins (Jazwinska et al., 2003), promote tube elongation in the *Drosophila* tracheas (Dong et al., 2014) and, despite molecular differences, in the *C. elegans* vulva (Cohen et al., 2020). While the structural integrity of most lumens relies on the hydration and rigidity of hydrophilic ECM components such as polysaccharides and glycoproteins, lumen patency can also be achieved through active surface-tension reduction. A prime example is the pulmonary alveolus. Here, the ECM is replaced by lung surfactant – which consist of 80% lipids, mostly phosphatidyl choline, together with hydrophobic surfactant proteins – showing that hydrophobic layers can also prevent lumen collapse (Pranty et al., 2026).

On the basal side, the viscoelastic and structural properties of the fibrous and networked ECM (Fig. 1A,B) impose physical constraints that prevent lumen expansion (Camacho-Gomez et al., 2022; Elosegui-Artola et al., 2023; Enemchukwu et al., 2016). Mechanical equilibrium between ECM-anchored epithelial cells and expanding luminal pressure ensures the maintenance of luminal architecture. Dynamic ECM remodeling is therefore crucial, as it alters material properties and releases biomolecules, notably for branching morphogenesis (Loganathan et al., 2020).

Together, these studies using molecular and cellular approaches have elucidated key aspects of polarity and lumen initiation. However, lumenogenesis involves the coupling of epithelial mechanics with lumen fluid hydraulics (Camelo and Luschnig, 2021; Dasgupta et al., 2018; Torres-Sanchez et al., 2021); therefore, understanding lumen morphogenesis requires an integrated physical framework that accounts for feedback between luminal forces and epithelial responses.

## Physical principles of lumenogenesis

**Fig. 2.**
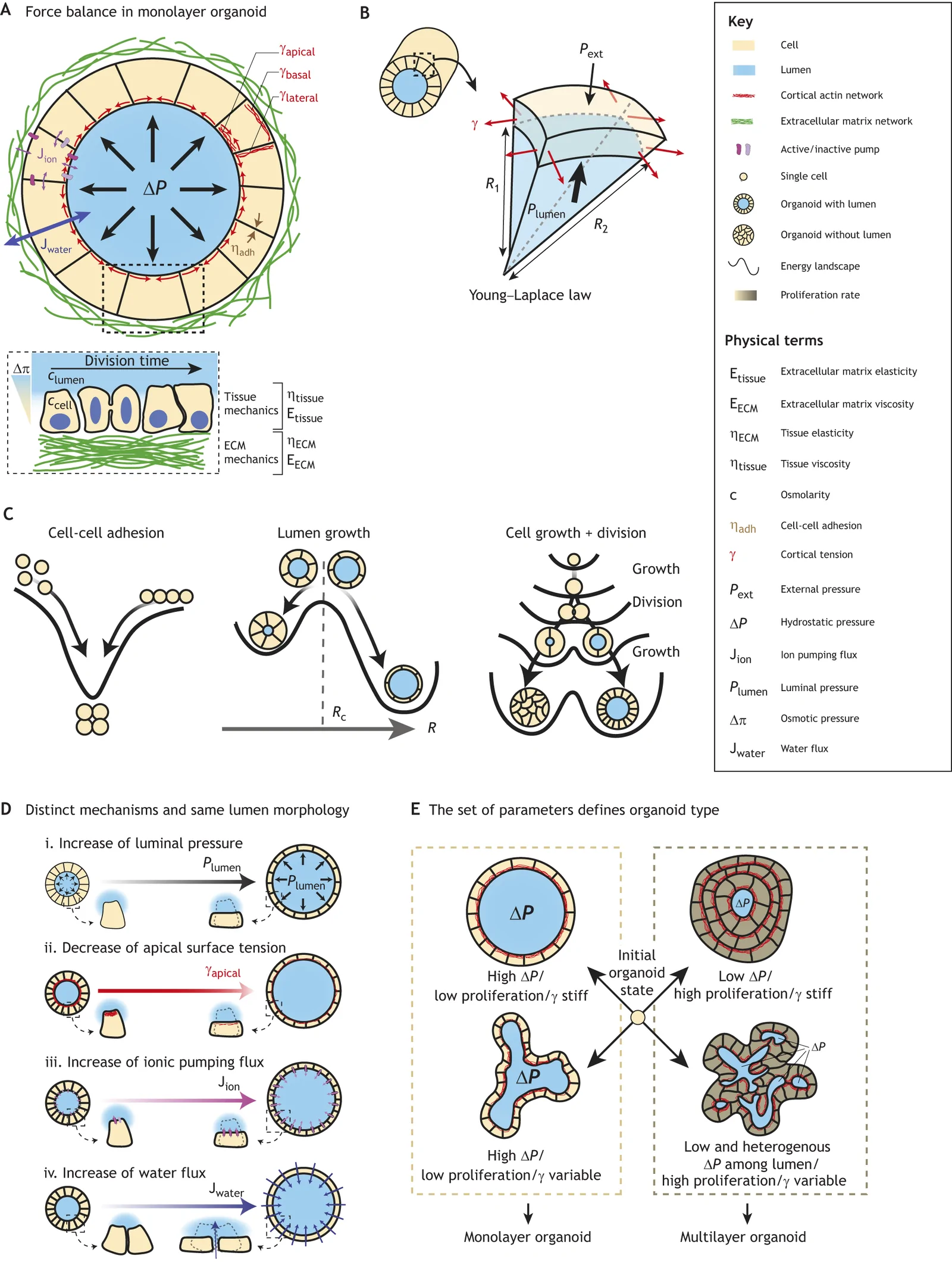
**Physical principles governing lumen formation and tissue morphology.** (A) A lumen surrounded by an epithelial cell layer is shaped by the balance between luminal pressure and mechanical resistance from the tissue and the ECM layers. Ion pumping (J_ion_) generates a gradient of ions across the monolayer and an osmotic pressure difference (ΔΠ). Water can enter through a flux (J_water_), which generates a hydrostatic pressure difference, Δ*P*, that can expand the lumen. This hydrostatic pressure is opposed by cortical tensions at the apical, basal and lateral cell surfaces, respectively (γ_apical_, γ_basal_ and γ_lateral_), by cell–cell adhesion, n_adh_, and by the elastic and viscous properties of both the tissue and the ECM. The detailed inset illustrates how cell division time and tissue osmolarity contribute to the evolving mechanical state of the organoid. Osmotic pressure triggers the generation of hydrostatic pressure, which is faced by the cell monolayer and might, in turn, increase its mass by proliferating. Timescales of increase in luminal volume, cell proliferation, tissue and ECM viscosities contribute to determining the lumen shape. (B) Young–Laplace law applied to the curved epithelial/luminal interface. The pressure difference across the tissue layer depends on cortical tension and curvature according to the Young–Laplace equation (Box 1). Here, *R*_1_ and *R*_2_ are the two principal radii of curvature of the curved interface. They originate from the fact that a three-dimensional surface can bend along two independent directions. For a spherical lumen, the two radii are equal, whereas for an irregular or elongated lumen, they may differ locally. (C) Effective energy-landscape view of lumen and tissue organization. The emergence of organoid architecture can be represented as transitions along energy landscapes. Cell–cell adhesion favors the assembly of isolated cells into cohesive clusters. Lumen growth can be described as a transition from small unstable lumina to larger stable lumina once a critical lumen radius, *R*_c_, is reached. Cell growth and division progressively reshape the landscape by increasing cell number and tissue mass, allowing the system to evolve toward different organoid morphologies, depending on the balance between adhesion, lumen expansion and proliferation. (D) Distinct physical mechanisms can generate similar final morphologies. Starting from the same initial organoid state, several independent mechanisms can promote lumen expansion and lead to a similar high-lumen occupancy phenotype with a large lumen and a stretched cell monolayer. These causes include: an increase in luminal pressure, *P*_lumen_; a decrease in apical cortical tension, γ_apical_; an increase in ion pumping flux, J_ion_; or an increase in water influx, J_water_. (E) Qualitative prediction of organoid morphology from a combination of physical parameters. The final organoid type depends on the combined effects of lumen pressure, proliferation rate and epithelial mechanical properties. High and homogeneous Δ*P*, together with low proliferation, favor lumen expansion and the formation of monolayered organoids. When cells divide, they tend to orient tangentially and the lumen remains spherical (top left). If local surface tension varies, then local curvature will vary following the Young–Laplace law (bottom left). With low Δ*P*, cell proliferation can occur radially and multilayered organoids can appear in particular in the context of a large and homogeneous surface tension (top right). With heterogeneous pressure between lumina, high proliferation and variable cortical mechanics favor multilayered and multi-luminal architectures (bottom right). For clarity in E, high Δ*P* is indicated by bold text, whereas low Δ*P* is indicated by regular text, instead of using pressure arrows (as in the previous panels). The schematics outlined on the left summarize parameter combinations associated with monolayer organoids, while the schematics outlined on the right summarize conditions associated with multilayer organoids.

From a physical perspective, lumen morphogenesis is governed by a force balance between cells, the ECM and luminal fluid (Fig. 2A). In a simple scenario, a difference in osmotic pressure between the lumen and the ECM associated to pumping activity triggers a potential passage of water. This generates a difference in hydrostatic pressure between the lumen and the ECM. The surface tension of the cell layer opposes the lumen expansion together with a resistance of the surrounding ECM. In a more elaborated version, timescales can be introduced for cell dynamics within the tissue as well as for reorganization of the ECM. In this context, effective viscosities can be measured and the interplay between tissue and ECM dynamics can be computed and tested experimentally. These physical rules are captured by specific equations and, together with calibrations of parameters (Table 1 and Box 1), solutions for shapes of lumen can be predicted and physical mechanisms can be tested.

Box 1. Equations that help understand lumen morphogenesis **Luminal hydrostatic pressure**

The luminal hydrostatic pressure (Δ*P*) is counteracted by effective tissue tension (γ), which incorporates the elasticity of monolayered or multilayered cell sheets and the ECM. This force balance is described by the Young–Laplace equation (Fig. 2B and Eqn 1), where *R*_1_ and *R*_2_ represent the principal radii of curvature in mutually orthogonal directions. For a tubular lumen, these radii differ between the axial and circumferential directions, whereas for a spherical lumen, they are equal (*R*_1_=*R*_2_). If the luminal pressure rises to a point where the induced tissue tension exceeds the intercellular adhesion force (η_adh_), cell-cell junctions break, potentially leading to lumen rupture.

Young–Laplace law:
(1)

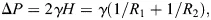
where Δ*P* is the hydrostatic pressure difference between inside and outside lumen, γ is the epithelial tension and 
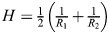
 is the mean curvature, where R_1_ and R_2_ represent the principal radii of curvature in orthogonal directions. For sphere with a radius *R*, mean curvature is H=(1/*R*).

**Lumen dynamics (see Fig. 2A)**

The dynamics of lumen volume are determined by the net rate of water influx (Eqn 2), which is the sum of three components: J_water_ (J_w_) [the aquaporin-mediated influx proportional to the apical surface area A_l_, water permeability λ_water_ and the pressure difference (Δπ–Δ*P*)]; J_leak_ (the fluid leakage through cell junctions); and J_cross_ (a minor water flow coupled with ion influx currents).
(2)

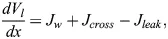
where *V_l_* is the lumen volume, *J_w_* is osmotic water flow mainly occurring via aquaporins, *J_cross_* is the water flow across paracellular junctions due to the cross-coupling effect with ion influx currents, *J_leak_* is the paracellular leak driven by the pressure gradient *J_w_*=*A_l_*λ*_water_*^(ΔΠ–Δ*P*)^, *A_l_* is the area of apical surface, λ*_water_* is the water permeability, ΔΠ is the osmotic pressure difference and Δ*P* is the hydrostatic pressure difference.

Solvable free energy for lumen, tissue and the ECM in the continuous model (Fig. 2C):
(3)

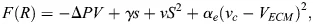
where *R* is the mean radius of curvature of the lumen, *V*=(4π/3)*R*^3^ is the lumen volume and *S*=4π*R*^2^ is the apical area. The term *S*^2^ represents the increase in surface elasticity due to stretching, while *α_E_* (*V_C_* − *V_ECM_*)^2^ represents the bulk elasticity of the ECM. If both are absent, *R* is convex upward, thereby the radius satisfying the Laplace's law (i.e. *R_c_*=2γ/Δ*P*) is unstable (see Fig. 2C). One of these nonlinear terms suffices to stabilize the lumen radius. New force balance becomes Δ*P*=2γ′/R with an effective surface tension γ′(*R*), which is increasing with *R*, where *V_ECM_* is the actual volume of ECM and *v*_c_ is the equilibrium volume of ECM.

**Table 1.** Physical parameters and perturbation methods

Physical parameter (units)	Physical meaning	Cellular origin	Spatial scale	Measurement methods*	Measured values	Perturbation methods^‡^
c (osmolarity) (mol/m^3^)	Osmolite concentration	Ions pumps and transporters	Cell and tissue	Osmometer (Krens et al., 2017)	Interstitial fluid (zebrafish embryo) at 250 mol/m^3^ (Krens et al., 2017)	Osmotic shocks (Chan et al., 2019)
ΔΠ (osmotic pressure) (Pa)	Solvent pressure driven by concentration difference between inside the lumen and the ECM	Osmolarity, ions pumps and transporters, water channels (aquaporin), polarized compartments (apical/lateral and basal), cytoplasm (regulation of the current leaks), junctional permeability, paracellular permeability and tight junctions	Cell and tissue	Double emulsion droplet sensors (Vian et al., 2023)	Interstitial fluid (zebrafish embryo) at ∼700 kPa (Vian et al., 2023)	Perturbations of ion channels Forskolin/CFTR functional assays Claudin-binding toxin cCPE (Lee et al., 2026) Genetic perturbations of tight junctions (Mukenhirn et al., 2024) Chemical perturbations of tight junctions and claudins (Chan et al., 2019)
J_ion_ (ionic pumping flux) (mol/s/m^2^)	Flux of ions secreted by cells and leaking across the cell layer	Ion pumps and transporters	Tissue	Volume change method after osmotic shocks (Latorre et al., 2018)	MDCK cysts and domes at 0.04-0.14 μmol/cm^2^ h (Latorre et al., 2018)	Chemical perturbations of ion pumps (Mosaliganti et al., 2019) Genetic perturations of transporters (Deng et al., 2013) Electric field perturbations (Shim et al., 2024)
λ (water permeability) (m/s/Pa)	Water permeability across a cell layer (linked to the water flux, or J_W_)	Junctional permeability, paracellular permeability and tight junctions	Cell and tissue	Volume change measurements (Gin et al., 2010; Timbs and Spring, 1996) or through coherent anti-Stokes Raman scattering (Yu et al., 2013)	MDCK cysts at ∼2.1 10^−10^ mm/h/kPa (Gin et al., 2010)	Indirect perturbation via cell-cell adhesion properties (Chan et al., 2019; Lu et al., 2025) TJ/claudins mutants and perturbations (Chan et al., 2019) Aquaporin mutations (Wang et al., 2019; Yu et al., 2013)
Δ*P* (hydrostatic pressure) (Pa)	Difference in pressure between inside the lumen and the ECM	Cell and tissue mechanical properties	Cell and tissue	Transepithelial laser cut (Lee et al., 2026; Mukenhirn et al., 2024)	MDCK cysts at 100-300 Pa (Lu et al., 2025; Mukenhirn et al., 2024) and pancreatic organoids at 10-30 Pa (Lee et al., 2026)	Injection of fluid in the lumen (Lu et al., 2025; Yang et al., 2019; Bebelman et al., 2023)
				Pressure probe (Chan et al., 2019; Yang et al., 2019) (Narayanan et al., 2020)	MDCK cysts at 100-300 Pa (Yang et al., 2019), MDCK at 37 Pa (Narayanan et al., 2020), blastocysts at 200-1500 Pa (Chan et al., 2019) and zebrafish inner ear at 100-300 Pa (Mosaliganti et al., 2019)	
γ (surface tension of the cell layer) (Pa.m or N/m)	Energy cost per unit surface area	Cell cortical tension coupled to cell-cell adhesion and tight junctions	Tissue	Micropipette aspiration and Laplace law inference (Lu et al., 2025) (Dumortier et al., 2019)	Blastocysts at 500-1000 pN/mm (Dumortier et al., 2019) and MDCK cysts and domes at 3-10 mN.m^−1^ (Lu et al., 2025) (Latorre et al., 2018)	Cell-cell adhesion properties (Chan et al., 2019; Lu et al., 2025) Cell cortical properties (Chan et al., 2019; Lu et al., 2025; Shim et al., 2024) Change of ECM stiffness (Perez-Gonzalez et al., 2021)
				Micropressure probe and Laplace law inference (Chan et al., 2019)	Blastocysts at 6 nN/μm (Chan et al., 2019)	
				Traction force microscopy (Perez-Gonzalez et al., 2021)	Mouse intestinal organoids (2D organoids – traction vector field magnitude) at 200 Pa (Perez-Gonzalez et al., 2021)	
				Atomic force microscopy	MDCK cysts at 5 mN/m (Perez-Tirado et al., 2025)	
E_Tissue_ (Young modulus of the cell layer) (Pa)	Elasticity of the cell layer	Cell cortical tension coupled to cell-cell adhesion and tight junctions	Tissue	Atomic force microscopy (Shen et al., 2017)	MDCK cysts at ∼8 kPa (Shen et al., 2017)	Indirect perturbations such as osmotic shocks, lumen volume change and cell contractility change (Shen et al., 2017)
η_Tissue_ (viscosity of the cell layer) (Pa.s)	Viscosity of the cell layer	Coupling between cell-cell adhesion, tight junctions and cell cortical tension	Tissue	Micropipette aspiration (Perros et al., 2024)	Hydra at 2.10^6^ Pa.s (Perros et al., 2024)	
E_ECM_ (elasticity of the ECM) (Pa)	Elasticity of the ECM	Polymerization and polymer binding	Tissue	Rheological measurements (shear rheometer)	Matrigel at ∼200 _Pa_(3)	Different mix of biopolymers (Gimenez et al., 2017) Dilutions (Klaas et al., 2016) Light activation of polymerization for patterning (Yavitt et al., 2023)
				Optical tweezers	Matrigel at ∼200 Pa (Borries et al., 2020)	
				Atomic force microscopy	Collagen I gels E at ∼100-1000 Pa (Gimenez et al., 2017)	
η_ECM_ (viscosity of the ECM) (Pa.s)	Viscosity of the ECM	Polymerization and polymer interactions	Tissue	Rheological measurements (shear rheometer)	Depends on the shear rate (.s^−1^): Matrigel at 100 Pa.s at a shear rate of 0.1 s^−1^ (Zeiringer et al., 2023)	
				Optical tweezers	Effective viscosity; useful to compare conditions. (Borries et al., 2020)	
				Atomic force microscopy	Effective viscosity; useful to compare conditions (Liu et al., 2024)	
η_Lum_ (viscosity of the lumen fluid) (Pa.s)	Viscosity of the luminal fluid	Secretion of proteins in the lumen and water viscosity	Tissue	Tracking of particles in the lumen and the Stokes-Einstein equation (Lee et al., 2026)	Pancreatic organoids at∼2 mPa.s (Lee et al., 2026)	
				Fluorescence correlation spectroscopy (Mukenhirn et al., 2024)	MDCK spheroids at 5 mPa.s (Mukenhirn et al., 2024)	
γ (acto-myosin cortical tension) (N/m)	Coarse-grained force generated within the acto-myosin cortex by molecular motors on a polymerizing actin gel	Actin turnover, actin-binding proteins, myosin motors and binding with the cell membrane	Cell	Laser ablation of the cell cortex (an indirect measurement)	Proof of contractility (extension/contraction) (Barai et al., 2025) and recoil velocity to compare contractility of cells or tension anisotropies (Perez-Gonzalez et al., 2021; Saha et al., 2016; Guyomar et al., 2026;	Myosin contractility perturbations (calyculin, Y-27632, ML-7 and blebbistatin) (Barai et al., 2025) (Lu et al., 2025; Mukenhirn et al., 2024), actin polymerization (latrunculins, cytochalasins and branched polyamines) (Vasquez et al., 2021; Lu et al., 2025; Guyomar et al., 2026)
				Optical tweezers, atomic force microscopy and micropipette aspiration (direct measurements)	for reviews, see Roca-Cusachs et al., 2017; Salbreux et al., 2012; Villeneuve et al., 2025) at ∼10^3^ pN.μm^−1^	Optogenetic tool to modify acto-myosin contractility (Krueger et al., 2025; Lu et al., 2024) and myosin mutants (Maitre et al., 2016)
η_adh_ (cell-cell adhesion force) (N)	Adhesion forces of cell-cell interfaces	N- and E-cadherin binding	Cell	Atomic force microscopy and micropipette aspiration (destructive methods) Quantification of detachment force	Mouse embryo at ∼40-100 nN and rupture tension at 5 nN.mm^−1^ (de Plater et al., 2025)	Cadherin mutants (de Plater et al., 2025) and calcium chelation of cadherin adhesion (Lu et al., 2025)
				FRET sensor (non destructive method) (Borghi et al., 2012; Lagendijk et al., 2017; Narayanan et al., 2020)	Ratio, useful to compare conditions	
t_d_ (cell proliferation) (h)	How fast cells replicate	Cell cycle timing	Cell	Live imaging, BrdU/EdU staining and cell count	8-25 h (Milo and Phillips, 2015; Lee et al., 2026; Lu et al., 2025)	Aphidicolin (Lee et al., 2026; Lu et al., 2025)

*Physical parameters can be measured in biological systems but in a labor-intensive manner and not simultaneously. They allow the simulations to run in order to calculate and predict shapes of lumina and tissues.

^‡^Physical parameters can be experimentally modified to evaluate their contributions to the experimental responses, which can lead to changes in other physical parameters and feedback mechanisms.

^§^See Slater et al. (2018)

### Biophysical parameters of lumen biogenesis, growth, shape and maintenance

#### Mechanical force balance and the regulation of lumen growth and shape

Lumen formation emerges from the interplay between the biophysical and biochemical factors that shape tissues. An increase in lumen volume requires water influx through aquaporins or paracellular gaps (the space between neighboring cells) (Fig. 1B). This fluid transport is driven by an osmotic pressure gradient, which is generated by active ion pumps and channels. The resulting increase in luminal ion concentration elevates the luminal osmotic pressure, driving water influx. This influx raises the luminal hydrostatic pressure, exerting a perpendicular force on the apical surface. At the micrometer scale, inertial forces are negligible (Purcell, 1976). Therefore, the mechanical equilibrium of the lumen is determined by hydrostatic pressure, tissue elasticity, active forces and friction with the surrounding tissue and the ECM (Fig. 2A).

These physical forces must balance locally throughout the tissue (Fig. 2B and Box 1, Eqn 1). While the force balance for a simplified system consisting of a spherical lumen, surrounding tissue and the ECM can be solved analytically (Box 1), computer simulations are indispensable for modeling how a lumen and tissue architecture emerge from a single dividing cell (Fuji et al., 2022; Akiyama et al., 2018). Although various computational models exist, they share a common approach: they define an effective total energy of the system and solve the governing force balance equations. While living cells and tissues are inherently non-equilibrium systems and therefore lack a strict thermodynamic free energy, an effective energy can be formulated by combining tissue elastic energy, cell–cell adhesion energy and the mechanical work associated with lumen expansion (Fuji et al., 2022; Honda et al., 2008; Mombach et al., 2001). This formulation is highly advantageous because, in mechanics, forces arise from spatial gradients of energy: each contribution to the effective energy generates a corresponding force that either promotes or resists tissue deformation. The time evolution of the system can therefore be viewed as moving down an energy landscape toward a local minimum. Consequently, this allows the force balance equations to be derived automatically by calculating the energy gradients with respect to relevant variables, while also providing an intuitive, graphical understanding of the process (Fig. 2C). In simplified form (Box 1, Eqn 3), the total energy consists of contributions from lumen growth, cell–cell adhesion (where larger contact areas minimize adhesion energy, Fig. 2C), cell surface area and tissue–ECM interactions.

Next, the shape dynamics of lumina need to be computed (Box 1, Eqn 2). Initially, lumina are often irregularly shaped, and their subsequent growth is governed by competing physical effects. The core mechanism can be illustrated by considering a spherical lumen with a defined radius under a given hydrostatic pressure. If the tissue tension is assumed to remain constant regardless of lumen size, a critical radius emerges. Lumina with radii smaller than the critical threshold radius cannot overcome the opposing tissue tension and therefore collapse. Conversely, lumina exceeding this threshold grow continuously (Fig. 2C). The persistence of micro-lumina below the critical radius is thought to result from electrostatic repulsion generated by glycoproteins present on the apical surface. In biological tissues, rather than growing indefinitely until rupture, lumen expansion eventually saturates at a stable radius due to an increase in tissue tension at larger radii and elastic resistance from the ECM (Tanida et al., 2025; Bedzhov and Zernicka-Goetz, 2014; Dokmegang et al., 2021; Gin et al., 2010; Lu et al., 2025).

#### Energy-based models of lumen morphogenesis

The process of repeated cell division leads to lumen formation, which can also be captured into an energy landscape framework (Fig. 2C). In these models, cell growth is represented by an energy term that drives each cell to expand and increase its volume, and cell division is triggered once a cell reaches a threshold volume (Belmonte et al., 2016; Tanida et al., 2025). By introducing a tiny initial micro-lumen at the cleavage plane during each cell division cycle, the system automatically evolves according to the balance between cell mechanics, adhesion and luminal forces, ultimately progressing toward a configuration that minimizes the total energy. However, if the rate of cell division or growth is fast enough to exceed the rate at which luminal hydrostatic pressure can be established, these micro-lumina collapse and disappear (Tanida et al., 2025; Lu et al., 2025; Lee et al., 2026). Furthermore, stochastic fluctuations in the initial cell arrangement or the orientation of the division plane can drastically alter the fate of the lumen (Fig. 2C). To capture these dynamic behaviors, computational models are broadly categorized based on their geometrical representations – treating cells as either polyhedra or continuous surfaces – and whether their governing equations are solved deterministically or stochastically (Fletcher et al., 2017; Fuji et al., 2022).

Importantly, varying different combinations of parameters within this force-balance framework can yield similar lumen shapes (Fig. 2D). Starting from the same initial lumen volume, different perturbations can drive expansion toward a similar final lumen shape and size through distinct mechanisms, including increased luminal pressure, reduced apical surface tension, altered ion pumping flux or increased water flux. Although these perturbations generate similar changes in luminal shapes, their underlying causes are distinct (Tanida et al., 2025; Lee et al., 2026). Changes of luminal volume associated to distinct mechanisms underscore the need for quantitative measurements of physical parameters underlying lumen morphogenesis (Table 1; Fig. 2D).

#### Mechanical constraints and lumen tissue architecture

Distinct combinations of mechanical parameters lead to different tissue organizations and lumen dynamics, revealing potential mechanisms that regulate lumen shapes (Hannezo and Simons, 2018; Tanida et al., 2025). Based on the principle of force balance (Fig. 2E), tissue architecture emerges from the interplay between hydrostatic pressure, proliferation and surface tension. When hydrostatic pressure is high, cells tend to be tangentially organized, favoring the formation of spherical lumen. In contrast, low hydrostatic pressure enables radial cell division and the development of a multilayered system (Tanida et al., 2025). Furthermore, surface tension modulates these behaviors by locally resisting hydrostatic pressure-driven expansion. Under conditions of high pressure and low proliferation, strong surface tension stabilizes spherical cysts, whereas spatial variations in surface tension, which leads to high hydrostatic pressure and low proliferation, can generate local curvature differences and departures from spherical symmetry. Conversely, low pressure with high proliferation promotes multilayered structures when surface tension is sufficiently strong. Spatial heterogeneities in pressures can further produce multi-lumen morphologies and curved multilayered architectures when cell proliferation levels are high (Tanida et al., 2025). Together, these simplified mechanical rules provide a framework for designing experiments aimed at testing mechanisms for morphogenesis, supported by quantitative measurements of key parameters (Table 1) along with theoretical models and simulations.

### Interplay of molecular determinants and physical parameters underlying lumenogenesis

The physical parameters of lumen morphogenesis (Fig. 2) correspond directly to underlying cellular and molecular structures (Fig. 1B and Table 1). At each cell interface, the acto-myosin cortex drives mechanical tension (Latorre et al., 2018; Saha et al., 2016). Adhesion complexes and their specific connections to the networks of actin, microtubule and intermediate filaments regulate the local mechanical properties of the actin cortex at each apical-lateral-basal side (Li et al., 2016; Guyomar et al, 2026). Specifically, actin filaments are anchored to ezrin at the apical membrane (facing the lumen and rich in microvilli), to the ECM on the basal side through integrin-mediated adhesion, to cadherins at lateral adherens junctions, as well as to tight junctions that seal the apical surface (Fig. 1Bii) (Sun et al., 2025; Tokuda et al., 2009). Additionally, desmosomes (laterally) and hemidesmosomes (basally) connect to intermediate filaments, which provide tensile strength and interact with actin filaments (Fig. 1B) (Latorre et al., 2018; Zuidema et al., 2020).

Fluid dynamics also shape the lumen. Ion pumps, channels and transporters – together with tight junctions and aquaporins – mediate the change in concentration across cellular compartments and drive osmotic pressure (Fig. 1B) (Dasgupta et al., 2018; Datta et al., 2011; Indana et al., 2024). Finally, the molecular nature of these adhesions dictates the overall force balance; for instance, the cytoskeleton assembly triggered by interacting laminin (a major ECM protein) differs from that of fibronectin (another predominant ECM protein) (Geiger et al., 2001), directly altering the mechanical equilibrium.

Qualitatively, these cellular and molecular changes can be used to predict changes in lumen shape. For example, an increase in the ECM crosslink density raises its elasticity (encoded by the Young modulus). Assuming all physical parameters remain constant, this increase in matrix resistance restricts luminal volume during inflation. Conversely, a decrease in apical resistance mediated by actin depolymerization lowers surface tension, leading to an increase in the luminal volume (Fuji et al., 2022; Tanida et al., 2025; Guyomar et al., 2026; Indana et al., 2024).

## Approaches to integrate molecular, physical and cellular parameters of lumenogenesis

### Computational simulations

**Fig. 3.**
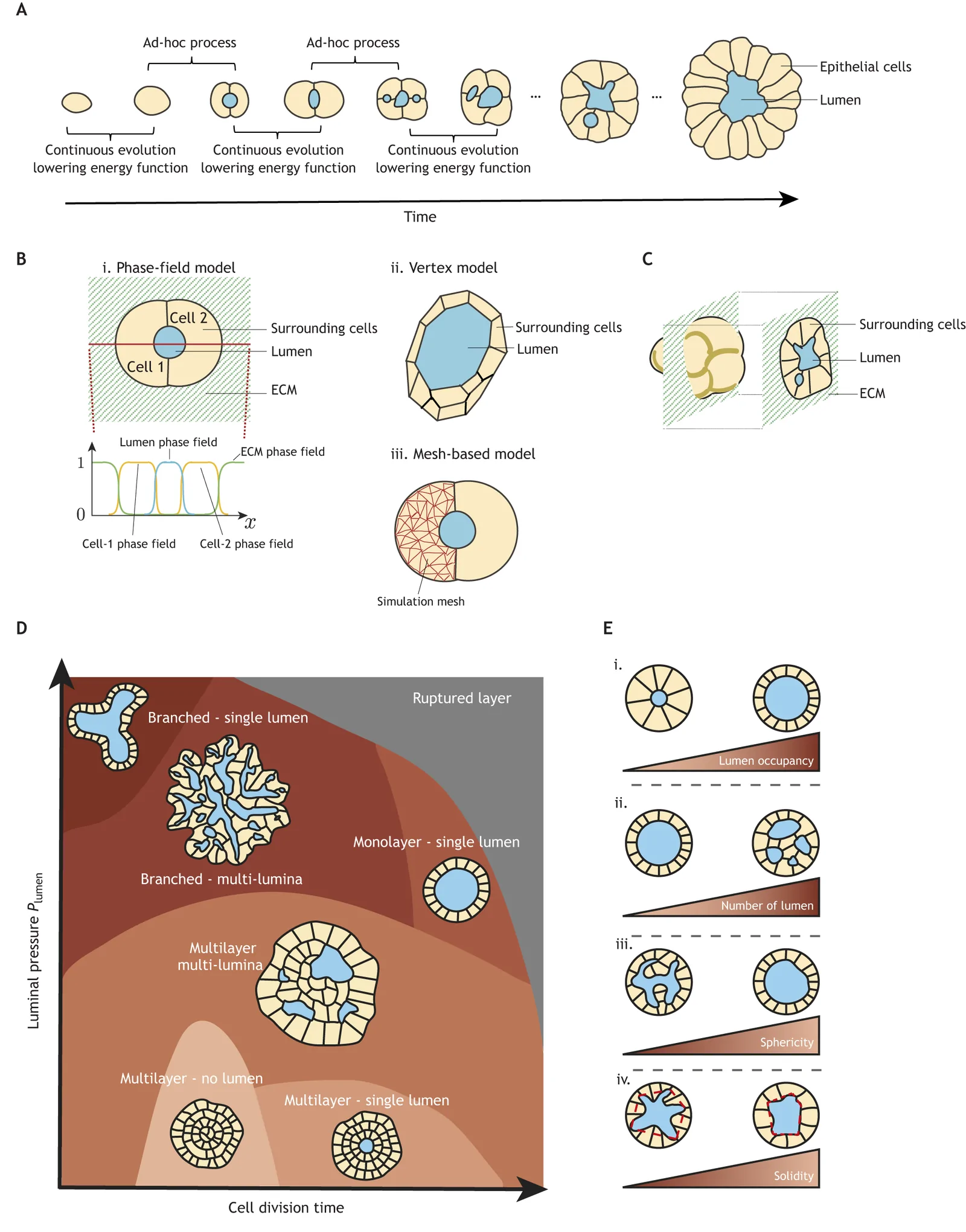
**Numerical simulations and quantitative analysis of lumen and tissue.** (A) Computational simulations allow the calculation of lumen and cell evolution over time and can predict the lumen morphology emerging autonomously after the growth. The simulations are usually carried out by combining the autonomous continuous time-evolution following the dynamics that gradually lower energy function with the ad-hoc processes that implement the features not described in the energy function. What features cannot be given in the energy function, or should be treated as ad-hoc processes, are dependent on the models, which provides the advantage of each model explained in the main text. This diagram particularly exemplifies the simulation of the phase-field model of Tanida et al. (2025), wherein cell division and new microlumen creation are the ad-hoc processes, whereas lumen fusions (or fissions) can be autonomously predicted following the evolution lowering energy function. (B) Examples of outcomes that can be obtained from numerical simulations using different models. (i) Phase-field model, wherein the shape of each object (lumen, each cell and, if necessary, ECM) are represented by the artificial fields called phase fields; yellow and blue curves at the bottom represent the fields associated with each cell and their lumen, respectively, while the green curves represent the ECM. (ii) Vertex model, wherein the interfaces between the elements are given by the straight edges in 2D and flat planes in 3D. (iii) Mesh-based model, wherein the shapes of some elements, such as each cell, are divided by the meshes with appropriate mechanical properties. (C) With most of the frameworks, 3D simulations are also available. This diagram particularly shows the case of the phase-field model, where 3D simulations can be performed assuming the third-directional stacks of 2D sections. (D) Phase diagram of simulated tissue and lumen morphologies as a function of luminal pressure, *P*_lumen_, and cell division time. Each colored region indicates the morphology predicted by the model for a given combination of luminal pressure and proliferation dynamics, with cell division time increasing from left to right and luminal pressure increasing from bottom to top. Representative tissue cross-sections illustrate the corresponding phenotypes: multilayered tissues without a lumen, multilayered tissues with a single lumen, multilayered tissues with multiple lumina, branched tissues with multiple lumina, branched tissues with a single lumen, monolayered tissues with a single lumen and ruptured epithelial layers. This map therefore links model parameters to predicted tissue architecture and provides a qualitative framework to understand how changes in luminal pressure and proliferation rate shape lumen organization. (E) Quantitative descriptors used to compare simulated lumen morphologies. Lumen organization is quantified using four complementary measurements: (i) lumen occupancy, corresponding to the fraction of the tissue volume occupied by luminal space; (ii) number of lumina, distinguishing single-lumen from multi-luminal structures; (iii) sphericity, measuring how close the lumen shape is to a round geometry; and (iv) solidity, measuring how compact or branched/irregular the lumen contour is. Together, these descriptors convert the qualitative morphologies shown in D into measurable features, allowing lumen shape transitions across the parameter space to be compared quantitatively between theory and experiments.

Theoretical models (as discussed in the section ‘Mechanical force balance and the regulation of lumen growth and shape’) provide a quantitative framework for predicting lumen dynamics across different parameter regimes (pressure, tension and material moduli) and for guiding experimental design. To implement the equations numerically, a lumen is generally treated as a quasi-cell whose expansion or shrinkage is driven by osmotic pressure, while intercellular adhesion contributes to stabilizing connective cellular tissue organization. Simulations typically begin with a fixed number of cells and define rules governing cell growth and division, based on specific values for growth speed, proliferation rate or cell division timing. In many models (Belmonte et al., 2016; Runser et al., 2024), the cell division plane is oriented either perpendicular to the long axis or randomly; more-advanced approaches additionally incorporate spindle mechanics and mechanical inputs from neighboring cells (Akiyama et al., 2018). Simulations then evolve the coupled cell–lumen system over time by iteratively updating geometries to decrease the defined energy function, while factoring in physical and cellular parameters, such as adhesion strength and cell cortical tension. Simultaneously, cell growth and division are implemented in a model-dependent manner (Fig. 3A). In many computational frameworks, hydrostatic pressure is not explicitly introduced but can be calculated implicitly in the following ways: at mechanical equilibrium, the hydrostatic pressure exerted by connective cells surrounding the lumen is balanced with lumen osmotic pressure (Box 1); hydrostatic pressure can also be autonomously determined by the tension and geometry of the connective cells surrounding the lumen so that it satisfies the force balance equation of the Young-Laplace law (Box 1). Together, the models provide a mechanistic framework for linking intracellular processes and tissue-scale morphogenesis (Fig. 3B).

A few examples of such morphodynamic computational frameworks include particle models (Akiyama et al., 2018), agent-based models, scaling models (Gunnarsson et al., 2024), coarse-grained continuous models (Chan et al., 2019; Lu et al., 2025), cell vertex models (Fig. 3Bii) (Fletcher et al., 2014; Honda et al., 2004; Lange et al., 2025; Nagai and Honda, 2001; Okuda et al., 2015a, 2013), the Cellular Potts model (Graner and Glazier, 1992; Hirashima et al., 2017), mesh-based models (Fig. 3Biii) (Okuda and Hiraiwa, 2023; Runser et al., 2024; Tanaka et al., 2015; Torres-Sanchez et al., 2022; Vetter et al., 2024) and multicellular phase-field models (Fig. 3Bi) (Akiyama et al., 2018; Nonomura, 2012; Tanida et al., 2025).

Each computational framework has its advantages and limitations that determine which biophysical questions it is best suited to address (Fuji et al., 2022). To illustrate these differences, we compare the basic principles, advantages and limitations of three representative modeling approaches next.

#### Phase-field models

Phase-field models describe cells and lumen as continuous spatial fields that evolve via energy minimization processes reflecting physical interactions. Owing to advances in spatial field-based mathematical constructions, phase-field models can naturally capture smooth shape changes, and lumen fusion and growth (Fig. 3A,Bi). Biophysical interactions and constraints are incorporated directly into the energy function. This approach has been applied to simulate lumenogenesis in epithelial cysts (Akiyama et al., 2018) and organoids (Guyomar et al., 2026; Tanida et al., 2025), where each cell and the entire lumen are represented by separate fields (Fig. 3B). A major advantage of phase-field models is that lumen fusion and deformation emerge naturally from the equations, without requiring additional manually designed fusion rules. Furthermore, in phase-field models, the ECM and additional biological variables, such as protein distributions, can be easily incorporated into the energy function (Fig. 3B) (Lu et al., 2025). However, because the model must calculate these variables at every point in space, simulations are computationally demanding, particularly in 3D. Therefore, they remain feasible only if system sizes or sample numbers are limited (Akiyama et al., 2018; Guyomar et al., 2026; Tanida et al., 2025).

#### Vertex models

Vertex models represent cellular tissues as polygons in 2D or polyhedra in 3D that share vertices and edges (Fig. 3Bii) (Fuji et al., 2022). Cell shapes change through vertex movements driven by biophysical forces related to optimum cell size, adhesion and cortical tension. In practice, the temporal evolution of these vertices is usually calculated via the gradient descent of a potential energy function encoding the corresponding effects. Furthermore, implementing topological rearrangements, specifically, the cell-cell junction remodeling known as T1 transitions, is crucial. These transitions represent neighbor exchanges between adjacent cells or between cells and the lumen, which are commonly observed during tissue remodeling and lumen morphogenesis. Without such rearrangements, lumina cannot change their topology through fusion or fission, making T1 transitions essential for lumenogenesis simulations. Biologically, these events correspond to local cell intercalation processes that allow tissues to relax mechanical stress and reorganize their structure. Vertex approaches have been widely used to model epithelial morphodynamics and 3D lumina (Eiraku et al., 2011; Odell et al., 1981; Okuda et al., 2015b, 2013, 2018a,b; Yu et al., 2024), including blastocyst lumen formation (Honda et al., 2008), organoids (Rozman et al., 2020), thyroid gland lumenogenesis (Gonay et al., 2021) and pancreatic ducts (Messal et al., 2019). In the standard vertex model, cell boundaries are represented by straight edges or flat faces, making it difficult to recapitulate curved lumen geometries, such as a single lumen sandwiched by only two cells. Additionally, topological rearrangements are not automatically captured by the energy-minimization framework and require additional ad-hoc rules (Fuji et al., 2022). However, because only vertex positions need to be updated in such cases, vertex models are usually more computationally efficient than multicellular phase-field models.

#### Meshwork models

Another approach to simulating lumenogenesis is to represent the cell surface as a meshwork composed of many small mechanical elements (Fig. 3Biii). This includes continuum mechanical descriptions of the cell surface, which are typically discretized into meshes for computational simulations (Bächer et al., 2021; Borja da Rocha et al., 2022; Okuda and Hiraiwa, 2023). Some models of this approach are available as open-source software and have been shown to simulate lumen morphology in epithelial cysts and mouse prostate organoids (Runser et al., 2024). Compared with vertex models, mesh-based approaches can represent complex and curved lumen geometries more naturally. At the same time, they are often computationally less demanding than phase-field models, particularly for 3D simulations (Fig. 3C). However, unless the mesh is appropriately ‘re-meshed’ during the simulation, the discretized mechanical elements can introduce artificial elasticity, making it difficult to accurately represent the fluid-like behavior of the cell membrane or cytosol (Fuji et al., 2022).

Regardless of which computational framework is used, all these modeling approaches can predict which multicellular morphologies – large-scale structural organizations adopted by lumina – emerge for given sets of control parameters such as adhesion strength, hydrostatic pressure, proliferation rate or cortical tension (Table 1). These parameter-dependent outcomes can be represented as phase diagrams (Fig. 3D), which map the emergence of different morphological states with respect to changing parameters. The resulting morphology types can then be classified using quantitative indices, such as lumen occupancy, the number of lumina, lumen sphericity and/or lumen solidity (Fig. 3E).

All models are essentially simplifications and therefore have limitations in their predictive power (Schwayer and Bruckner, 2023). For example, the effect of the surrounding matrix may be neglected in some cases but included in others (Fuji et al., 2022). Many models are implemented in 2D instead of 3D, which may limit the relevance if the system is not rotationally invariant (Hannezo and Simons, 2018). Finally, theoretical frameworks often rely on dimensionless parameters – where characteristic quantities such as time, length and force are normalized. Consequently, mapping these unitless values to equivalent experimental parameters with physical units is not always straightforward (Lenne and Trivedi, 2022; Prost et al., 2015).

### Comparison between computational models and experiments

To quantitatively compare physical model predictions with experimental observations, key mechanical parameters such as hydrostatic pressure, osmotic pressure, cortex tension, adhesion strength and material elasticities need to be measured in the biological systems of interest (Table 1). Once experimentally determined, these parameters can be incorporated as inputs into computational models to simulate the evolution of tissue geometry and topology during lumen formation and growth (Chan et al., 2019; Dumortier et al., 2019; Li et al., 2018; Xue et al., 2025).

Model predictions of lumen dynamics can be evaluated against experimental results using quantitative morphological parameters, such as lumen volume, occupancy, number of lumina, sphericity or solidity (Fig. 3E). To test the predictive power of different models, parameters can be systematically changed to create phase diagrams describing the resulting morphological response. These theoretical predictions can then be compared with experimental perturbations that affect the corresponding physical parameters in real tissues, thereby providing a test of model validity (Table 1). In practice, however, perturbations by targeting molecular actuators via drugs or genetics are often indirect, which can affect several parameters simultaneously, complicating interpretations. For example, activation of myosin leads to an increase in contractility, which may also secondarily affect cell–cell, cell–ECM adhesion and other physical parameters through force-dependent stabilization of adhesion (Balaban et al., 2001; Riveline et al., 2001). Similarly, functional inactivation of the tight junction proteins ZO1/ZO2 can lead to reduced hydrostatic pressure and increased cortical tension in MDCK cysts (Mukenhirn et al., 2024), which in turn increase the apical surface tension. In line with the force balance argument presented above (Fig. 2D), this simultaneous drop in hydrostatic pressure and rise in surface tension accounts for a small lumen phenotype. Additionally, the role of the ECM in defining surface tension distribution was evaluated (Li et al., 2016) using minimal assays with hepatocytes on controlled protein coatings in both 2D and 3D environments. These studies demonstrated that lumen localization and morphology can be predicted and controlled by quantitative adjustments to cell adhesion and geometrical constraints.

If key physical parameters are unknown or not directly measurable, models for understanding lumen morphogenesis can also be used to determine these values by fitting simulations to experimental data through calibration. However, if models are complex and if there are too many unknown parameters, overfitting can be a significant problem. The predicted phenotypes may not always reflect the actual causes of physical changes and this may limit the capacity of the model to make robust statements (Mangan et al., 2017).

## Feedback between lumen mechanics and morphogenesis

**Fig. 4.**
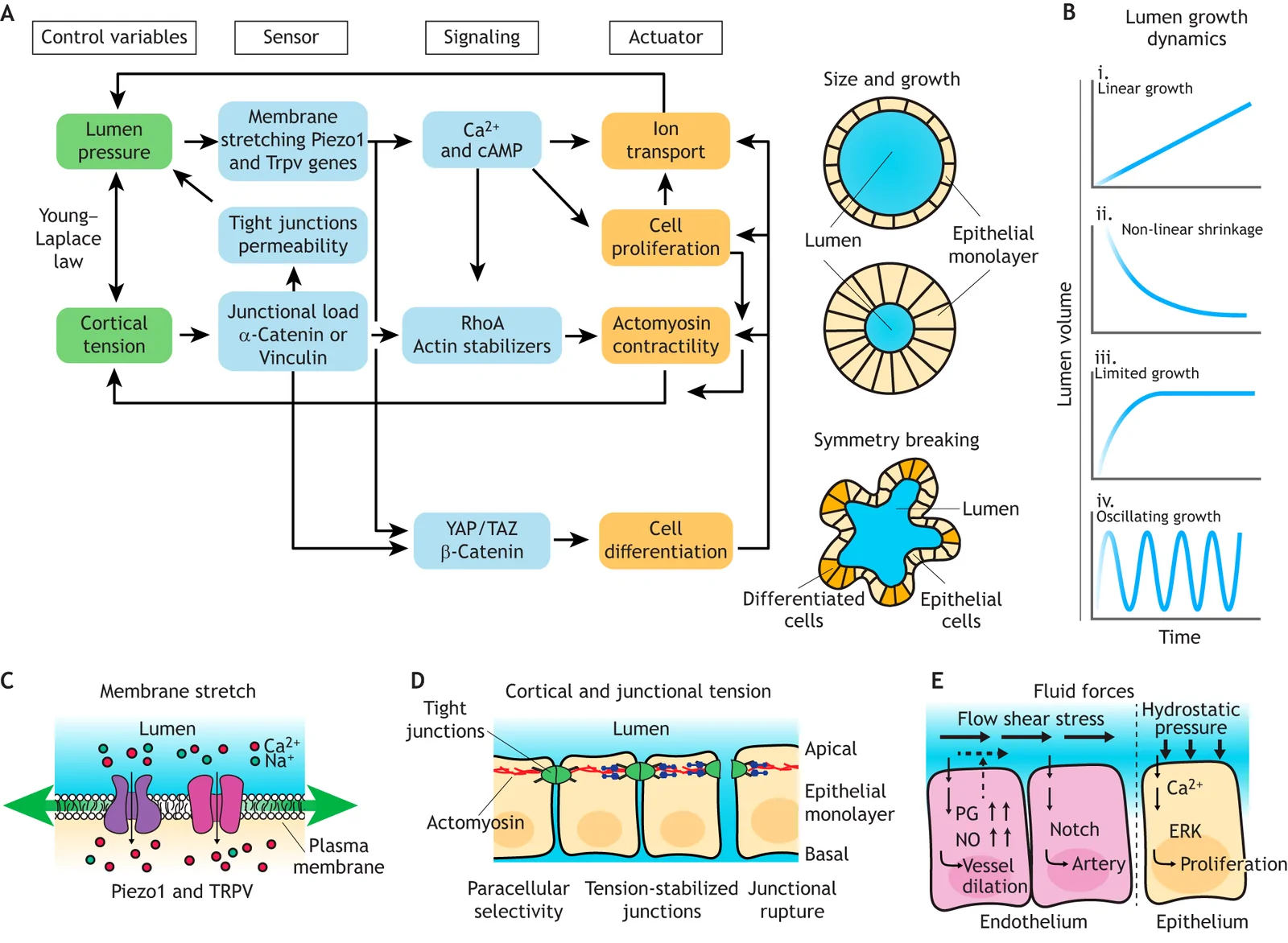
**Fluid mechanics-driven feedback controls lumen morphogenesis.** (A) Feedback diagram showing how control variables such as hydrostatic lumen pressure and cortical tension (green) are controlled by active biological cellular processes such as ion transport, actomyosin contraction, cell divisions and cell differentiation (orange). The control variables and actuators are connected in a feedback loop via sensing and signaling processes (light blue). The force balance of lumen pressure and cortical tension controls the size and growth of the lumen. The Young–Laplace law (Box 1) links the tension, lumen diameter and hydrostatic pressure, leading to large lumina with thin walls at high pressure or low tension and small lumina with thick walls at low pressure or high tension. Symmetry breaking into complex shapes involves positive feedback between cell differentiation and physical control variables. (B) Feedback control can produce differential volume growth (*y*-axes) dynamics through time (*x*-axes). (i) Linear growth, (ii) shrinking, (iii) growth to a stable set-point and (iv) volume oscillations. (C-E) Depiction of cellular modules involved in sensing fluid mechanics. (C) Membrane stretching is sensed by mechanosensitive channels (Piezo1 and TRPV) transporting predominantly calcium ions (Ca^2+^), but also other anions such as sodium (Na^2+^). (D) Cortical and junctional tension are sensed by mechanosensitive scaffold proteins (e.g. α-catenin or vinculin), components of adherens or tight junctions linking cells (green). Together they control paracellular selectivity in response to tension. They are reinforced by tension but beyond a certain force, junctional rupture and leakage are observed (E). Fluid forces such as shear from flow or pressure act perpendicularly to the epithelium, affecting intracellular responses. Flow is sensed by the apical membrane leading to secretion of prostaglandin (PG) and nitric oxide (NO), whereas pressure can control Ca^2+^ levels and downstream ERK signaling. The dashed arrows represent a negative-feedback loop, triggered by high shear stress, that leads to vessel dilation and, in turn, results in a lowered shear stress.

Minimal models often treat pressure, permeability or tension as fixed parameters in lumen morphogenesis. In reality, these quantities are dynamically coupled, with mechanical forces directly regulating cellular behaviors. For example, ion transport can be regulated by membrane tension through mechanosensitive ion channels and transporters (Choudhury et al., 2022; Gudipaty et al., 2017), paracellular permeability adapts to hydrostatic pressure through junction remodeling (Phillips et al., 2008; Tokuda et al., 2009; Tokuda and Yu, 2019), and actomyosin contractility responds to sustained strain through mechanochemical feedback (Chan et al., 2019; Dasgupta et al., 2018; Mukenhirn et al., 2024) (Fig. 4A). Incorporating mechanochemical feedback – where mechanical forces alter cellular biochemistry, and biochemical signals in turn regulate a the mechanical state of a cell (Aström and Murray, 2008) – is therefore essential to capture lumen dynamics and requires coupling between physical parameters in theoretical models. Yet, the molecular and cellular mechanisms that implement feedback between mechanical forces and active cellular processes are often incompletely understood.

### Feedback underlying lumen growth and stability

Feedback is important to control lumen growth towards a defined volume, surface and shape, and to provide robustness against perturbations. Negative feedback typically counteracts deviations and stabilize system states, whereas positive feedback can amplify perturbations and drive transitions or symmetry breaking. Notably, many lumen-forming systems such as hepatocytes, blastocysts (Chan et al., 2019; Niimura, 2003), *Hydra* (Fütterer et al., 2003; Kucken et al., 2008; Suzuki et al., 2023 preprint) and intestinal organoids (Farin et al., 2016; Sato et al., 2009; Tallapragada et al., 2021) exhibit oscillations in lumen volume during growth and maturation (Fig. 4B). Oscillations may not have a specific role here but are observed in feedback-controlled systems when delayed feedback causes repeated overshooting of the target state (Casani-Galdon and Garcia-Ojalvo, 2022; Novák and Tyson, 2008). Thus, lumen oscillations suggest feedback mechanisms that regulate lumen growth and size.

Dasgupta et al. (2018) showed that stable lumen growth in bile canaliculi emerges from negative feedback: ion pumping drives lumen expansion, but increasing pressure stretches cells, reducing cell-cell interface length and enhancing paracellular leakage, which releases pressure. Concurrently, actomyosin tension increases under strain, further stabilizing lumen size without requiring junctional rupture. In the initial formation of bile canaliculi, tight junctions are immature and therefore the paracellular leakage simply depends on the length of the cell-cell interface. However, in mature epithelia tissues, tight junctions can be almost completely impermeable to ions and water or leaky to certain ions in a tissue-specific manner (Gunzel et al., 2026; Phillips et al., 2008; Tokuda et al., 2009; Tokuda and Yu, 2019) (Fig. 4A,D). Tight junctions are also mechanically integrated into the apical cortex and regulate cortical tension via scaffolding proteins such as zonula occludens proteins (Citi, 2019). Furthermore, perturbations of tight junctions in MDCK cysts have strong phenotypes for lumen formation, ranging from promoting growth when transepithelial permeability is slightly increased to inhibiting growth when the tight junctions are completely lost (Mukenhirn et al., 2024). Feedback regulation involving adherens junctions also contributes to regulating tissue tension, notably in the vascular system under pressure where specialized actin-based protrusions, called junctional fingers, have been described (Kotini et al., 2022).

An alternative model explaining lumen volume oscillations involves the burst–heal oscillator (Ruiz-Herrero et al., 2017). In this model, lumen inflation increases tissue tension until the epithelial barrier – including tight and adherens junctions – ruptures, causing rapid fluid and pressure release. Following this fast burst, cells reform their junctional barriers and a new cycle of lumen expansion can begin. This simple feedback model of hydraulically-gated paracellular pressure release effectively captures luminal size regulation and predicts both the amplitude and timescale of volume oscillations. Taken together, these studies support the view that lumen size control relies on feedback between hydrostatic pressure, paracellular permeability and mechanical tension of the actomyosin cortex. However, quantitative comparisons of lumen pressure, cortical tension and paracellular permeability across various systems are needed to establish the underlying mechanosensing pathways and to clarify whether this feedback mechanism dictates lumen growth dynamics in all systems where lumen oscillations are observed.

In addition to the above, cellular ion pumping is important to control lumen growth as it drives the osmotic movement of water. In secretory epithelia, the Na^+^/K^+^-ATPase (sodium–potassium pump) establishes transmembrane ion gradients by actively exporting Na^+^ and importing K^+^ across the basolateral membrane. These gradients provide the driving force for secondary transporters such as the sodium-potassium-chloride co-transporter 1, which accumulates chloride ions (Cl^−^) within epithelial cells. Cl^−^ is subsequently secreted into the lumen through apical channels, generating transepithelial osmotic gradients that drive water transport through aquaporins and paracellular pathways, thereby promoting lumen inflation and fluid accumulation (Frizzell and Hanrahan, 2012). Evidence for the importance of feedback regulation of epithelial fluid transport was shown in kidney cells, where changes in transepithelial pressure gradients affect the expression and localization of pumps and transporters in a cytoskeletal-dependent manner, affecting fluid flux. Polycystin 1 and polycystin 2 – the proteins forming a mechanosensitive calcium ion (Ca^2+^) channel mutated in autosomal dominant polycystic kidney disease – exhibit impaired pressure-dependent functions. However, to what degree additional mechanisms – such as membrane stretch and osmotic swelling – activate mechanosensitive channels such as Piezo1, TRPV4 and TRPM7 to regulate intracellular Ca^2+^ signaling and transporter activity remains an unanswered question (Fig. 4C) (Srivastava et al., 2020; Zhao et al., 2019).

### Feedback between pressure, proliferation and cell death

Mechanical forces generated by lumen pressure feed back on epithelial growth by regulating cell proliferation. During hydraulic lumen inflation, epithelial cells lining the lumen are stretched (Chan et al., 2019; Dasgupta et al., 2018; Gin et al., 2010; Ruiz-Herrero et al., 2017; Yang et al., 2021). Stretching of epithelial cells is a potent trigger for proliferation that has been observed in epithelial tissues across vertebrate systems, including mammals and zebrafish, and is mediated in part by the mechanosensitive ion channel Piezo1 (Fig. 4C,E) (Gudipaty et al., 2017; He et al., 2018; Pathak et al., 2014). Mechanical deformation of the membrane activates Piezo1-dependent calcium influx, which in turn stimulates downstream signaling pathways such as MEK/ERK and cell-cycle regulators, promoting mitotic entry (Fig. 4E) (Gudipaty et al., 2017). Cell stretching during lumen inflation thus provides potential feedback that drives stabilization of hydraulic pressure via cell proliferation (Fig. 4A,C). Pressure-induced lumen growth has been implicated in multiple developmental and pathological contexts, including cyst growth in epithelia and aberrant expansion in kidney cyst disease (Alcorn et al., 1977; Cerruti et al., 2013; Desmond and Jacobson, 1977; Gin et al., 2010; Lee et al., 2026; Tanida et al., 2025). Although a few molecular mediators of mechanical inputs are known, as exemplified above, detailed knowledge of full mechanochemical feedback loops is still awaited. Conversely, excessive cell compression or crowding can activate Piezo1-dependent pathways leading to cell extrusion or cell death, indicating that the same mechanosensory machinery can support distinct, sometimes even opposing, outcomes depending on the mechanical context (Eisenhoffer et al., 2012).

In addition to Piezo1 signaling, stretch-induced proliferation has been linked to β-catenin, YAP/TAZ and Hippo pathway activity (Aragona et al., 2013; Azzolin et al., 2014; Benham-Pyle et al., 2015). In the intestinal epithelium, mechanical strain promotes β-catenin-dependent transcription and cell-cycle entry (Benham-Pyle et al., 2015), while changes in cell spreading, cell density and cytoskeletal tension regulate the nuclear localization and activity of YAP/TAZ (Aragona et al., 2013; Azzolin et al., 2014). Since YAP/TAZ signaling molecules are negatively regulated by the Hippo pathway, these observations provide a mechanistic link between tissue mechanics and proliferative control. Together, these pathways enable epithelial tissues to translate pressure-induced deformation into changes in growth and tissue homeostasis.

### Feedback between hydraulics and electrical fields

Active ion pumping across polarized epithelia not only generates osmotic gradients but also produces transepithelial electrical fields. These electrical fields arise from asymmetric ion transport at apical and basal membranes, and can be further shaped by epithelial geometry, particularly in small or curved luminal structures (Duclut et al., 2019).

In addition, transepithelial electric fields generated by asymmetric ion transport can drive electro-osmotic flows within the narrow paracellular spaces (Saw et al., 2022). In such confined geometries, electrical currents passing through charged extracellular surfaces drag water along the paracellular pathway, thereby coupling ion transport directly to fluid flow (Bagnat et al., 2007; Fischbarg et al., 2017; Marbach and Bocquet, 2019; Sánchez et al., 2002). Since tight junctions control paracellular ion selectivity and electrical resistance across the epithelia, they are expected to be key regulators of these electrohydraulic couplings (Bagnat et al., 2007).

Recent experimental work further supports a direct role for bioelectric signals in lumen morphogenesis. Using epithelial cysts, Shim et al. (2024) demonstrated that externally applied electrical stimulation can induce lumen inflation and growth, a process termed electro-inflation (Shim et al., 2024). Their results suggest that electrical fields can modulate ion transport and fluid accumulation, thereby providing a mechanism through which bioelectric signals influence lumen size and tissue morphogenesis. These findings show that that electrical fields are not merely byproducts of epithelial transport but can themselves act as regulators of lumen dynamics.

At the timescales of tissue remodeling and morphogenesis (ranging from hours to days), additional electromechanical couplings may influence lumen dynamics. One example is membrane flexoelectricity, whereby membrane curvature induces electrical polarization and, conversely, electric fields can generate membrane bending. Theoretical models predict that flexoelectric stresses can effectively reduce or even reverse tissue surface tension, thereby promoting lumen expansion or collapse, depending on their orientation relative to fluid pumping (Duclut et al., 2021; Duclut et al., 2019). These models further predict that when electrical, hydraulic and mechanical forces oppose one another, stable lumen sizes or self-sustained oscillations can emerge without requiring epithelial rupture, in contrast to burst–heal oscillators driven by transient barrier failure (Duclut et al., 2021; Duclut et al., 2019; Ruiz-Herrero et al., 2017). Together, these studies establish electrical fields as active regulators of lumen morphogenesis through their coupling to hydraulic and mechanical forces.

### Feedback between fluid-generated forces and cell state

Fluid-generated forces such as shear stress and hydrostatic pressure not only shape lumen geometry but also influence cell identity and differentiation. In endothelial cells, shear stress induces the production of nitric oxide and prostaglandin, leading to vessel dilation and establishing a negative feedback loop that reduces shear levels (Fig. 4E) (Sriram et al., 2016). More pronounced fate changes occur during vascular development, where blood flow regulates arterial–venous identity through mechanotransduction pathways involving Notch signaling (Geudens et al., 2019; Jahnsen et al., 2015; le Noble et al., 2004; Moyon et al., 2001; Weijts et al., 2018).

Pressure- and flow-dependent regulation of cell state has also been observed in non-vascular contexts. In the mouse blastocyst, increasing hydrostatic pressure during lumen expansion over several hours reinforces tight junction maturation through vinculin recruitment, strengthening the ability of the trophectoderm epithelium to bear tension (Chan et al., 2019). Over longer developmental time spanning one or more cell cycles, luminal pressure has also been implicated in regulating asymmetric divisions of trophectoderm cells, thereby promoting the generation of inner cell mass cells, potentially through YAP-dependent mechanisms (van den Goor et al., 2024). These observations suggest that hydrostatic pressure can influence both the mechanical properties of the epithelium and subsequent cell fate decisions.

More recently, Zhang et al. reported that particle movements within the blastocyst lumen correlate with induction of Klf2, a marker of the inner cell mass, and proposed that fluid flows contribute to cell fate specification (Zhang et al., 2023). However, the interpretation that directed luminal flows act as the primary instructive signal remains speculative and preliminary, as perturbations of lumen dynamics can affect multiple aspects of blastocyst morphogenesis and cell fate determination. Thus, while the study supports a role for lumen-associated mechanical cues in developmental patterning, the specific contribution of luminal flow remains to be established.

In *Xenopus*, increased blastocoel pressure suppresses YAP nuclear localization and Wnt signaling, reducing neural crest differentiation, although a feedback mechanism linking these fate changes back to tissue mechanics or lumen dynamics has not yet been identified (Alasaadi et al., 2024). Similarly, elevated transmural pressure in developing mouse lungs promotes epithelial maturation, including increased surfactant and mucin production (Nelson et al., 2017). Because these changes modify surface tension and the rheological properties of luminal fluids, they may in turn alter lumen mechanics, providing a potential route through which pressure-induced changes in cell state feed back onto tissue morphogenesis.

Because multiple physico-chemical cues can be perturbed at the same time during lumen inflation (e.g. osmotic pressure, hydrostatic pressure, tissue stretch, tension, concentration of molecules, etc.), it is often difficult to disentangle which exact physical cue drives changes in cell state or differentiation. For example, lumen inflation in intestinal organoids has been associated with changes in stem cell fate and differentiation, including fetal-like regenerative programs and crypt patterning during morphogenesis, but the underlying mechanisms remain incompletely understood (Tallapragada et al., 2021; Xue et al., 2025; Yang et al., 2021). Similarly, amniotic fluid inhalation in the developing lung promotes differentiation of alveolar type I cells, a process that depends on luminal fluid accumulation and tissue distension, yet the specific mechanical or physicochemical cues responsible for this response remain unknown (Li et al., 2018).

Together, these examples illustrate that fluid-derived, specifically lumen-derived, forces can act as instructive signals that couple morphogenesis, mechanics and cell fate. In doing so, they place lumen formation within broader feedback networks that couple physical forces to tissue differentiation programs.

## Conclusions

Luminal cavities are not passive architectural features but dynamic mechanical compartments that shape tissue morphogenesis through hydraulic, electrical and biomechanical feedback. Integrating molecular cell biology with physical theory and quantitative modeling has revealed conserved principles governing lumen initiation, growth and stability across systems. However, major challenges remain, including improving synergies between biological and physical experiments and analysis. Currently, each parameter has to be measured individually, which requires a lot more effort than, for example, the multiparameter measures of single cell transcriptomes. Future work will require experimental developments towards the systematic measurements of physical parameters *in vivo*, as well as experimental perturbations to disentangle coupled feedback loops. Incorporating artificial intelligence and physics-informed models creates opportunities for higher throughput in analysis and predictions. Emerging stem cell-based models that deviate from spherical shapes, such as pancreas organoids and embryo models, will enable to address how asymmetries in lumen shape are created. Integrating engineering approaches with organoid systems will also enable the creation of boundary conditions that constrain epithelial shapes and external material properties, allowing researchers to test how these factors affect the lumen. In this respect, it remains challenging to incorporate mesenchymal populations into organoids to create more physiologically relevant epithelial boundaries. Advanced live imaging now offers opportunities to dynamically test theoretical predictions represented as multidimensional phase diagrams combining parameters. Moreover, linking cell differentiation and molecular states to physical and morphogenic outcomes is expected to become an active and important field, providing deeper insights into feedback mechanisms. A deeper understanding of how cavity-enclosed fluids instruct morphogenesis will not only clarify fundamental developmental processes but also inform regenerative medicine and disease modeling.
